# Damage‐Free Passivation of Ambipolar OECTs with Fluoropolymer Film for Enhanced Performance and Stability Toward Reliable Biosignal Processing

**DOI:** 10.1002/advs.77167

**Published:** 2026-09-02

**Authors:** Junyeong Yang, Bohyun Lee, Hanna Lee, Mingyu Kim, Changsoon Choi, Jung Ah Lim, Youson Kim, Sung Gap Im

**Affiliations:** ^1^ Department of Chemical and Biomolecular Engineering Korea Advanced Institute of Science and Technology (KAIST) Yuseong‐gu Republic of Korea; ^2^ Electronic and Hybrid Materials Research Center Korea Institute of Science and Technology (KIST) Seoul Republic of Korea; ^3^ GIST InnoCORE AI‐Nano Convergence Institute For Early Detection of Neurodegenerative Diseases Gwangju Institute of Science and Technology Gwangju Republic of Korea; ^4^ KAIST Graduate School of Semiconductor Technology KAIST Yuseong‐gu Republic of Korea; ^5^ Center For Quantum Technology Korea Institute of Science and Technology (KIST) Seoul Republic of Korea; ^6^ Department of Electrical and Computer Engineering Sungkyunkwan University (SKKU) Suwon Republic of Korea; ^7^ Division of Nano and Information Technology KIST School University of Science and Technology of Korea (UST) Seoul Republic of Korea

**Keywords:** ambipolar vertical OECT, biosignal processing, event‐based organic bipolar spiking encoder, fluoropolymer passivation, matrix‐domain nanostructure

## Abstract

Organic electrochemical transistor (OECT) is a promising building block for bioelectronic and neuromorphic applications due to its high transconductance and low‐voltage operation. However, microstructural instability and irreversible electrochemical degradation of the organic mixed ionic‐electronic conductors (OMIECs) are still challenging to achieve high performance of OECTs. Here, we present an ambipolar vertical OECT in which a fluorinated passivation layer is deposited on the OMIEC surface directly by solvent‐free initiated chemical vapor deposition (iCVD) method without damaging the channel layer. Vapor‐phase infiltration of the fluoropolymer layer followed by thermal annealing induces controlled nanoscale phase separation in the OMIEC layer, enabling efficient ion doping in both p‐type and n‐type modes. Consequently, fluoropolymer‐passivated ambipolar vertical OECTs (FPA‐vOECTs) exhibit higher transconductance, far enhanced transient response, and longer operational stability. In particular, the transconductance—a key figure of merit—increased from 12.7 to 73.7 mS in p‐type and from 4.1 to 21.4 mS in n‐type operation. Leveraging the enhanced performance of the ambipolar vertical OECTs, we also demonstrate, for the first time, a single‐channel bipolar event‐driven spiking encoder for neuromorphic bioelectronics, enabling electrocardiogram signal encoding and spiking neural network‐based arrhythmia classification with 90.5% accuracy.

## Graphical abstract

A damage‐free iCVD fluoropolymer passivation strategy is introduced for ambipolar vertical OECTs. Vapor‐phase infiltration and thermal annealing induce nanoscale phase separation in the OMIEC channel, enhancing overall device performance. A single‐channel bipolar event‐driven spiking encoder is also demonstrated for electrocardiogram signal encoding and spiking neural network‐based arrhythmia classification, achieving 90.5% accuracy.

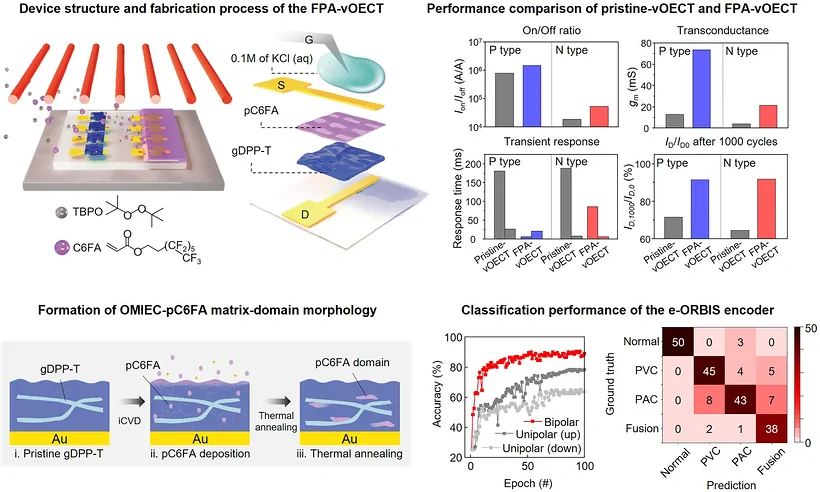

## 1 Introduction

Owing to their exceptionally high transconductance (> 10 mS), low operating voltage (< 1 V), mechanical flexibility, and good biocompatibility, organic electrochemical transistors (OECTs) are well suited for wearable and implantable electronics [1, 2, 3, 4, 5, 6]. Importantly, these advantageous characteristics allow OECTs to serve as versatile bioelectronic building blocks at biointerfaces, functioning as sensors [7, 8], signal amplifiers [9, 10], and encoding/processing elements [11, 12]. Despite remarkable progress in OECTs, further improvements in signal amplification, sensitivity, switching speed, and operational stability are still required for demanding bioelectronic applications and reliable real‐time interfacing with complex biological signals [13, 14]. High sensitivity and fast switching generally rely on rapid volumetric ion doping and dedoping of the channel from the electrolyte, together with efficient electronic transport within the organic mixed ionic‐electronic conductor (OMIEC) layer [15]. However, repeated volumetric doping and dedoping cycles often induce swelling‐driven microstructural changes and unintended redox side reactions of OMIECs, resulting in electrochemical degradation and operational instability [16, 17]. Therefore, mitigating the trade‐off between high charge transport performance and durable operation remains a critical challenge for OECTs.

Accordingly, significant research efforts have been devoted to improving the stability of OMIECs. Previous studies have primarily focused on the design and synthesis of new OMIEC materials, including novel conjugated backbone structures, donor–acceptor architectures, and side‐chain engineering strategies to enhance the ionic and electronic transport [18, 19, 20, 21]. While these material‐based approaches have achieved notable improvements in transconductance and switching speed, they often involve complex synthesis routes, limited material availability, and challenges in reproducibility and large‐scale manufacturing. As a result, it is still not trivial to translate such high‐performance materials into scalable and reliable bioelectronic devices. Besides synthesizing new materials, alternative strategies have been explored, including small‐molecule blending [22, 23, 24, 25, 26], bilayer‐stacked channels [27, 28], and surface modifications [29] (Table S1). However, these approaches frequently lead to poor long‐term electrochemical stability, unintended redox side reactions, or degraded transient response under repeated operation. These limitations highlight the necessity for distinctive OMIEC channel materials and processing strategies that are robust against electrical performance degradation while remaining compatible with simpler and more scalable fabrication processes [21, 30].

**FIGURE 1:**
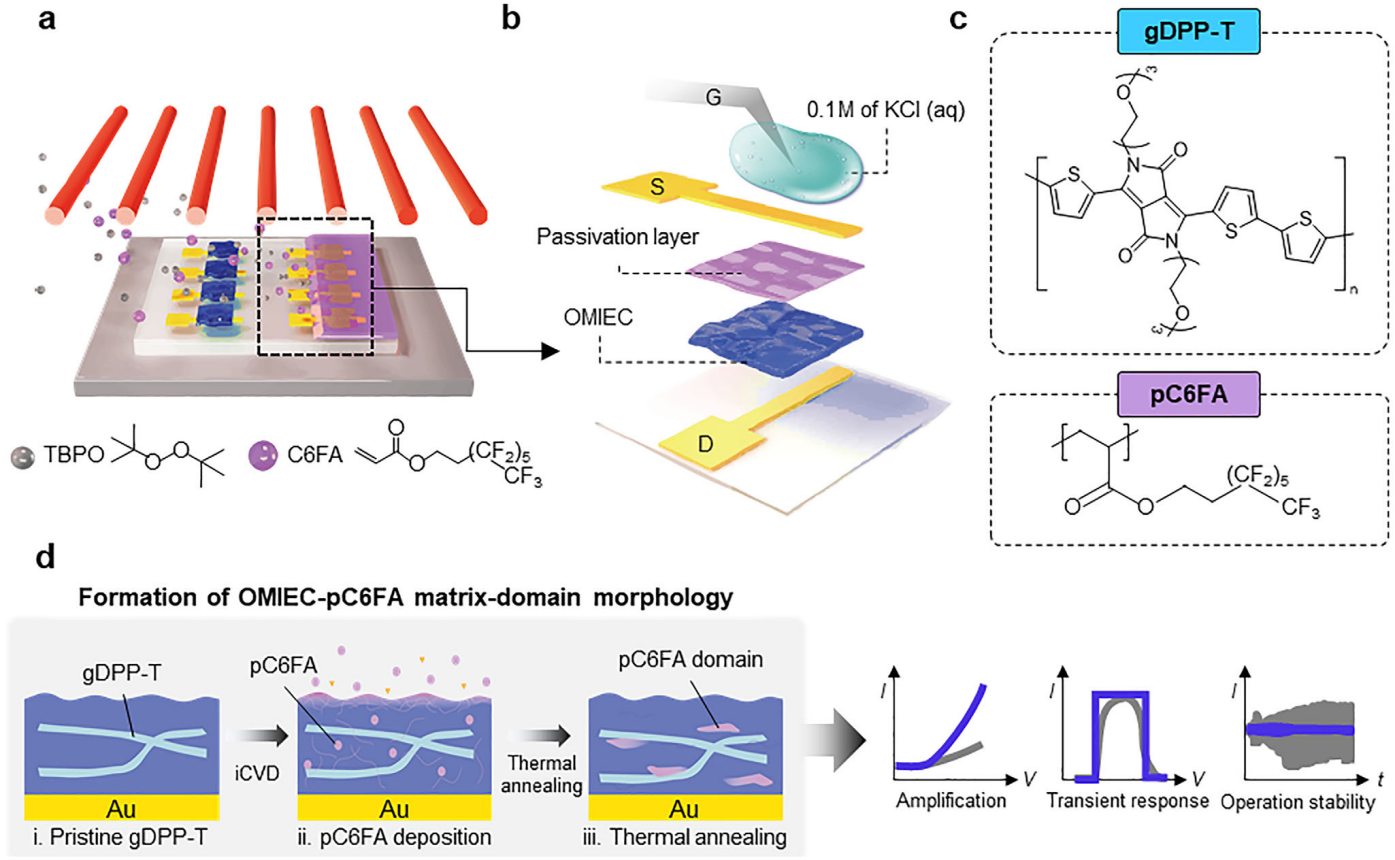
(a) Schematic illustration of the iCVD process for fluoropolymer‐based thin‐film passivation. (b) Device architecture and characterization of the FPA‐vOECT. (c) Chemical structures of the gDPP‐T semiconductor used as an OMIEC and pC6FA used as the passivation layer. (d) Schematic illustration of the process steps leading to the formation of the OMIEC‐pC6FA matrix‐domain morphology, employed as a novel strategy to improve the electrical characteristics, transient response, and operational stability of the OECT.

Herein, we propose a damage‐free, vapor‐phase passivation strategy based on initiated chemical vapor deposition (iCVD) as a novel approach for enhancing OECT performance and operational stability [31]. As illustrated in Figure 1a, the passivation layer is directly deposited onto the OMIEC surface via the iCVD process. Notably, unlike conventional surface‐coating approaches, polymer growth during iCVD inherently involves interchain diffusion into the underlying OMIEC layer, enabling the precise modulation of the bulk properties of the OMIEC, including film morphology and polarity. Building on this unique characteristic of the iCVD process, poly(2‐(perfluorohexyl)ethyl acrylate) (pC6FA), a fluoropolymer with highly polar C─F bonds, was selected as the passivation layer. This passivation strategy was implemented seamlessly in vertical organic electrochemical transistors (vOECTs) employing 2,5‐di(2,5,8,11,14‐pentaoxahexadecan‐16‐yl)‐3‐(5'‐methyl‐[2,2'‐bithiophen]‐5‐yl)‐6‐(5‐methylthiophen‐2‐yl)‐2,5‐dihydropyrrolo[3,4‐c]pyrrole‐1,4‐dione (gDPP‐T), an ambipolar OMIEC, as the channel material (Figure 1b,c). Ambipolar OMIECs enable both p‐type and n‐type operation within a single‐channel, which can simplify the design of OECT‐based complementary circuits and increase integration density [32, 33]. From a bioelectronics perspective, ambipolarity provides an additional advantage in that a single‐channel can detect and transduce signals associated with both cations and anions, thereby broadening the prospects of ambipolar OMIEC‐based devices for biosensing [33, 34]. Despite this potential, limited availability of high‐performance ambipolar OMIECs remains a critical bottleneck, hindering further advances in ambipolar OECTs and their complementary circuits. In this study, iCVD‐based passivation strategy is presented that simultaneously enhances the performance and operational stability of ambipolar OECTs, offering a viable pathway toward high‐performance, reliable bioelectronics. Through vapor‐phase passivation, iCVD‐deposited pC6FA infiltrates into the gDPP‐T layer to form an intermixed polymer network, which can further be regulated by a subsequent thermal annealing step to induce a fluorine‐rich matrix‐domain nanostructure, as schematically illustrated in Figure 1d. This morphological change organizes ion doping and dedoping pathways and suppresses redox side reactions, thereby enabling more efficient and stable ion doping in both p‐type and n‐type modes, driven by both cations and anions.

As a result, fluoropolymer‐passivated ambipolar vOECTs (FPA‐vOECTs) show substantial performance enhancements in both p‐type and n‐type operation. Specifically, the transconductance increases from 12.7 to 73.7 mS (p‐type) and from 4.1 to 21.4 mS (n‐type), while the turn‐on transient response decreases from 180.8 to 6.5 ms (p‐type) and from 188.1 to 85.9 ms (n‐type). In addition, current retention improves from 71.5% to 91.4% for p‐type operation and from 64.4% to 91.9% for n‐type operation, indicating enhanced operational stability. Consequently, whereas prior efforts to improve ambipolar OMIECs have largely relied on the design and synthesis of new materials, our approach demonstrates that vapor‐phase passivation of the widely used ambipolar OMIEC gDPP‐T can substantially enhance the device performance without damaging the channel layer nor requiring complex material design.

Finally, we apply the newly developed FPA‐vOECTs as neuromorphic spiking encoders suitable for AI‐driven wearable device applications. In previous studies, p‐type and n‐type semiconductors have typically been used separately to generate positive and negative spiking responses, which are then combined to construct the encoder [35, 36, 37, 38, 39, 40]. In contrast, owing to the intrinsic ambipolarity and high‐performance of FPA‐vOECTs, we demonstrate for the first time a bipolar event‐driven spiking encoder without the need to separately prepare and integrate distinct p‐type and n‐type OMIECs. This encoder was capable of reliable conversion of electrocardiogram (ECG) signals into bipolar spiking signals. Furthermore, the MIT‐BIH arrhythmia dataset [41] was encoded into two‐channel spike trains and subsequently used for training and inference with a spiking neural network (SNN), achieving a recognition accuracy as high as 90.5% for four‐class classification. Overall, this work demonstrates that a simple vapor‐phase passivation strategy can induce functional nanoscale morphology in OMIECs, leading to substantial improvements in OECT performance and supporting their application in neuromorphic bioelectronic systems.

## 2 Results and Discussion

### 2.1 Fabrication of FPA‐vOECT and Formation of Fluorine‐Rich Matrix‐Domain Nanostructures

The fabrication process of the FPA‐vOECT is illustrated in Figure S1, and the detailed procedures are also described in the Experimental Section. The key distinction between the FPA‐vOECT and a typical vOECT lies in the direct incorporation of the polymer passivation step prior to the deposition of the top electrode. The resulting device architecture and the chemical structures of the OMIEC channel material (gDPP‐T) and the passivation layer (pC6FA) are presented in Figure 1b,c, respectively. The optimized thickness of the pC6FA layer was 30 nm for FPA‐vOECT fabrication, yielding the highest transconductance (*g*_m_) among the investigated conditions (Figure S2 and see the Experimental Section for details). In this vertical geometry, the device dimensions are defined by a channel width (*W*) and thickness (*d*) of 100 µm, while the channel length (*L*) is approximately 150 nm.

The synthesis of pC6FA via the iCVD process was confirmed by Fourier‐transform infrared (FT‐IR) spectroscopy (Figure S3). The C═C vinyl peak of the C6FA monomer at 1635 cm^−1^ disappeared after iCVD polymerization, indicating effective radical polymerization of the C6FA monomer. In addition, characteristic absorption peaks at 1735 cm^−1^ for C═O stretching vibration and 1153 cm^−1^ for ─CF_2_‐CF_3_ bonds are well preserved in the pC6FA spectrum, confirming that the pC6FA retains the key functional groups of the C6FA monomer [42].

As mentioned in the introduction section, through the iCVD process, vapor‐phase C6FA monomers diffuse into the gDPP‐T polymer layer, followed by radical polymerization within the penetrated region to form pC6FA polymer in situ. As a result, an interpenetrating polymer network (IPN) forms at the gDPP‐T/pC6FA interface, which is fully consistent with previous observations [43, 44]. Subsequent thermal annealing increases the polymer chain mobility to promote the chain reorganization within the intermixed gDPP‐T/pC6FA layer, leading to the formation of a matrix‐domain nanostructure (Figure 1d).

**FIGURE 2:**
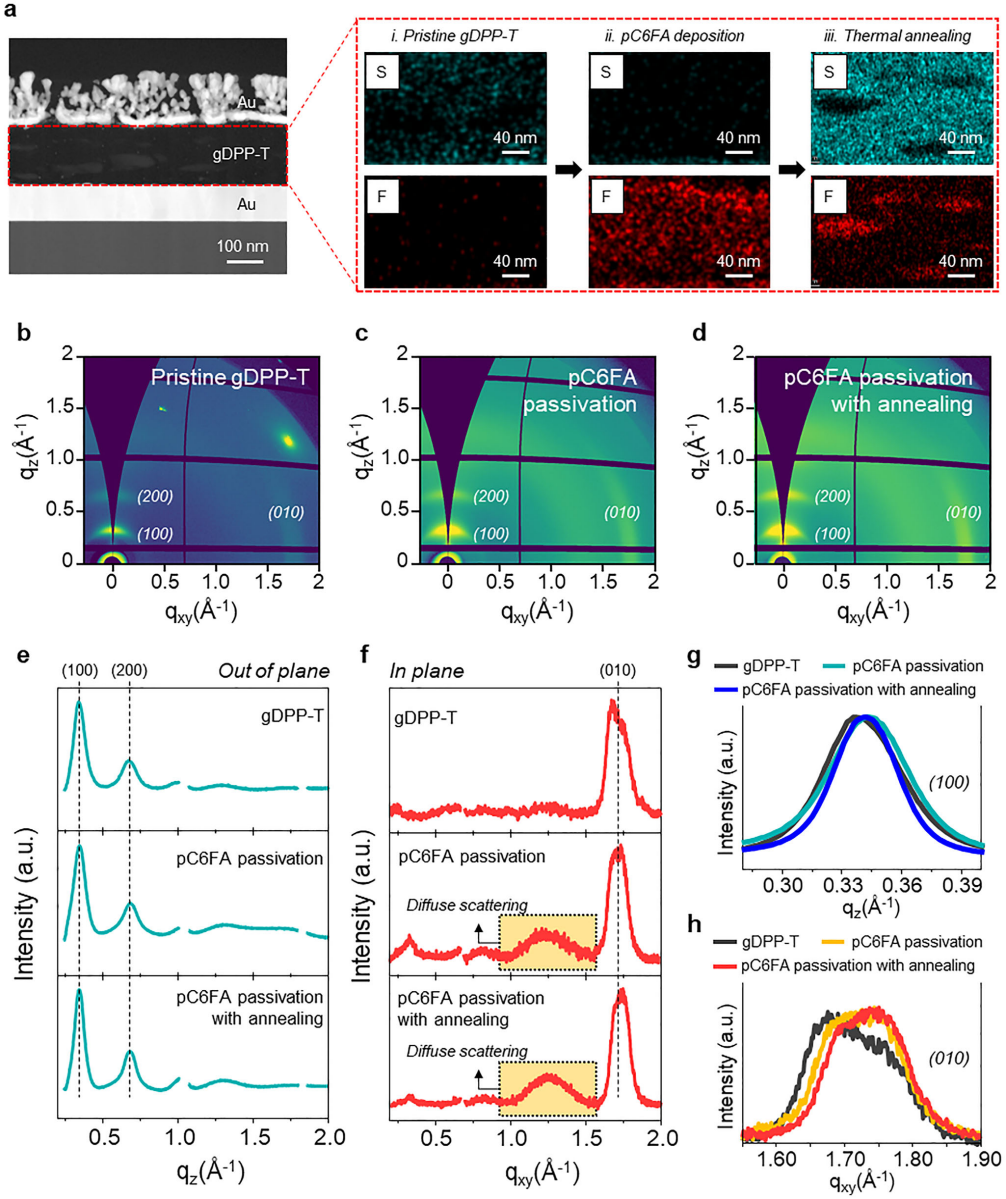
(a) The cross‐sectional TEM images and corresponding EDS scanning reveal the evolution of elemental distributions within the OMIEC layer at each processing step, confirming the formation of the domain morphology of pC6FA. (i) A pristine gDPP‐T channel exhibits undetectable fluorine signal within the OMIEC layer. (ii) After the iCVD process, fluorine signals originating from pC6FA are uniformly distributed throughout the OMIEC layer. (iii) Following thermal annealing, fluorine is locally aggregated within the sulfur‐rich OMIEC layer. GIWAXS 2D diffraction patterns of (b) pristine gDPP‐T, (c) pC6FA‐passivated gDPP‐T, and (d) thermally annealed pC6FA‐passivated gDPP‐T films. Corresponding 1D line profiles extracted along the (e) out‐of‐plane and (f) in‐plane directions. Magnified comparisons of the (g) (100) lamellar‐stacking peak and (h) (010) π‐π stacking peak for the three different samples.

This formation of the suggested nanostructure was monitored using cross‐sectional transmission electron microscopy (TEM) and corresponding energy‐dispersive X‐ray spectroscopy (EDS) elemental mapping (Figure 2a). The pristine vOECT channel exhibits a uniform sulfur distribution originating from the thiophene units of gDPP‐T, while no fluorine signal, corresponding to pC6FA, is detected (Figure 2a (i)). After the iCVD passivation, fluorine signals emerge throughout the gDPP‐T layer, indicating infiltration of pC6FA into the OMIEC matrix (Figure 2a (ii)). Following thermal annealing at 150°C for 30 min, the fluorine distribution becomes more pronounced and spatially reorganized within the thin channel layer, exhibiting clear segregation from sulfur‐rich regions, which supports the formation of fluorine‐rich domains embedded within the gDPP‐T matrix (Figure 2a (iii)). EDS line‐scan analysis of the elemental distribution further supported this domain–matrix structure, showing a complementary spatial variation between the sulfur and fluorine atomic fractions (Figure S4). The sulfur atomic fraction remained at approximately 20% in the gDPP‐T matrix region but decreased to nearly 0% in the pC6FA domain region, whereas the fluorine atomic fraction increased from approximately 3% in the gDPP‐T matrix region to approximately 10% in the pC6FA domain region. The resulting matrix‐domain nanostructure formed through iCVD passivation and thermal annealing was further corroborated by time‐of‐flight secondary ion mass spectrometry (ToF‐SIMS), confirming a spatially overlapped yet phase‐separated distribution of pC6FA and gDPP‐T in the final FPA‐vOECT structure (Figure S5). Also, the uniformity of the gDPP‐T/pC6FA matrix‐domain nanostructure formed after iCVD infiltration and thermal annealing was evaluated by cross‐sectional TEM analysis (Figure S6).

This structural formation was interpreted as the result of a combination of thermodynamic and kinetic factors. First, pC6FA is hydrophobic due to its highly fluorinated side chains [45], whereas gDPP‐T exhibits pronounced hydrophilicity arising from its ethylene glycol side chains. This difference in surface energy reduces the enthalpic driving force for mixing, thereby lowering miscibility and promoting phase separation in the gDPP‐T/pC6FA blended system [46]. In addition, pC6FA is a polymer with a low glass transition temperature (*T*_g_) of around −30°C [42], providing high chain mobility that facilitates the rearrangement and aggregation within the gDPP‐T matrix during the thermal annealing at 150°C for 30 min. These thermodynamic and kinetic characteristics are therefore considered to promote phase separation between pC6FA and gDPP‐T domains, leading to the formation of a matrix‐domain nanostructure.

To investigate the structural evolution of the gDPP‐T film induced by pC6FA incorporation and subsequent fluorine‐rich domain formation, additional grazing‐incidence wide‐angle X‐ray scattering (GIWAXS) characterization was performed. To track the effect of the passivation process on the molecular packing structure of the channel layer, three different sample conditions were prepared: pristine gDPP‐T, pC6FA‐passivated gDPP‐T, and thermally annealed pC6FA‐passivated gDPP‐T films with fluorine‐rich domain morphology.

The corresponding 2D GIWAXS patterns are presented in Figure 2b–d. According to the 2D diffraction patterns, the as‐coated pristine gDPP‐T film exhibited pronounced (100) and (200) lamellar stacking peaks along the out‐of‐plane direction together with a (010) π‐π stacking peak along the in‐plane direction, indicating that the pristine gDPP‐T film predominantly adopts an edge‐on molecular orientation (Figure 2b). Figure 2c,d further show that the (100), (200), and (010) diffraction peaks remain preserved even after the pC6FA passivation and subsequent thermal annealing processes, indicating that the penetration of pC6FA into the OMIEC film and the formation of fluorine‐rich domains do not significantly disrupt the intrinsic crystalline framework of gDPP‐T.

Figure 2e,f show the one‐dimensional line‐cut profiles extracted from the 2D diffraction patterns along the out‐of‐plane and in‐plane directions, respectively. After pC6FA passivation, an additional broad scattering feature emerged in the intermediate *q* region (*q* = 1.0–1.5 Å^−1^) in the in‐plane direction. This broad feature is attributed to diffuse scattering originating from disordered intermolecular correlations within amorphous or free‐volume‐rich intermixed regions formed during the pC6FA infiltration process, suggesting that pC6FA is preferentially distributed within less‐ordered regions of the semiconductor film rather than within the highly ordered crystalline domains [47, 48].

Figure 2g,h compare the (100) and (010) diffraction peaks under the three different sample conditions. Compared with pristine gDPP‐T, both the pC6FA‐passivated and thermally annealed pC6FA‐passivated films exhibited slight shifts of the (100) and (010) peaks toward higher q values (Figure S7a). Specifically, the (100) peak shifted from *q_z_* = 0.336 to 0.342 Å^−1^, corresponding to a reduced lamellar spacing from 18.71 to 18.36 Å, while the (010) peak shifted from *q_xy_* = 1.68 to 1.745 Å^−1^, corresponding to a reduced π‐π stacking distance from 3.75 to 3.60 Å. In addition, the full width at half maximum (FWHM) values of both peaks decreased after the subsequent thermal annealing process (Figure S7b), indicating sharper diffraction features and more compact molecular packing. Furthermore, compared with the thermally annealed pristine gDPP‐T film (Figure S7c), the thermally annealed pC6FA‐passivated film exhibited a more pronounced higher‐*q* shift of the (100) lamellar peak, while the (010) π‐π stacking peak showed a similar peak position. Considering that pC6FA is preferentially distributed within amorphous or less‐ordered regions rather than within the crystalline domains, these results imply that the formation of fluorine‐rich domains may primarily influence the surrounding lamellar or side‐chain‐rich packing regions without significantly perturbing the intrinsic π‐π stacking framework of gDPP‐T.

In addition, atomic force microscopy (AFM) measurements reveal negligible changes in surface roughness before and after pC6FA deposition (Figure S8), indicating that the formation of the internal nanostructure does not induce any significant topographical modification of the OMIEC surface.

### 2.2 Electrical Characterization of FPA‐vOECT

**FIGURE 3:**
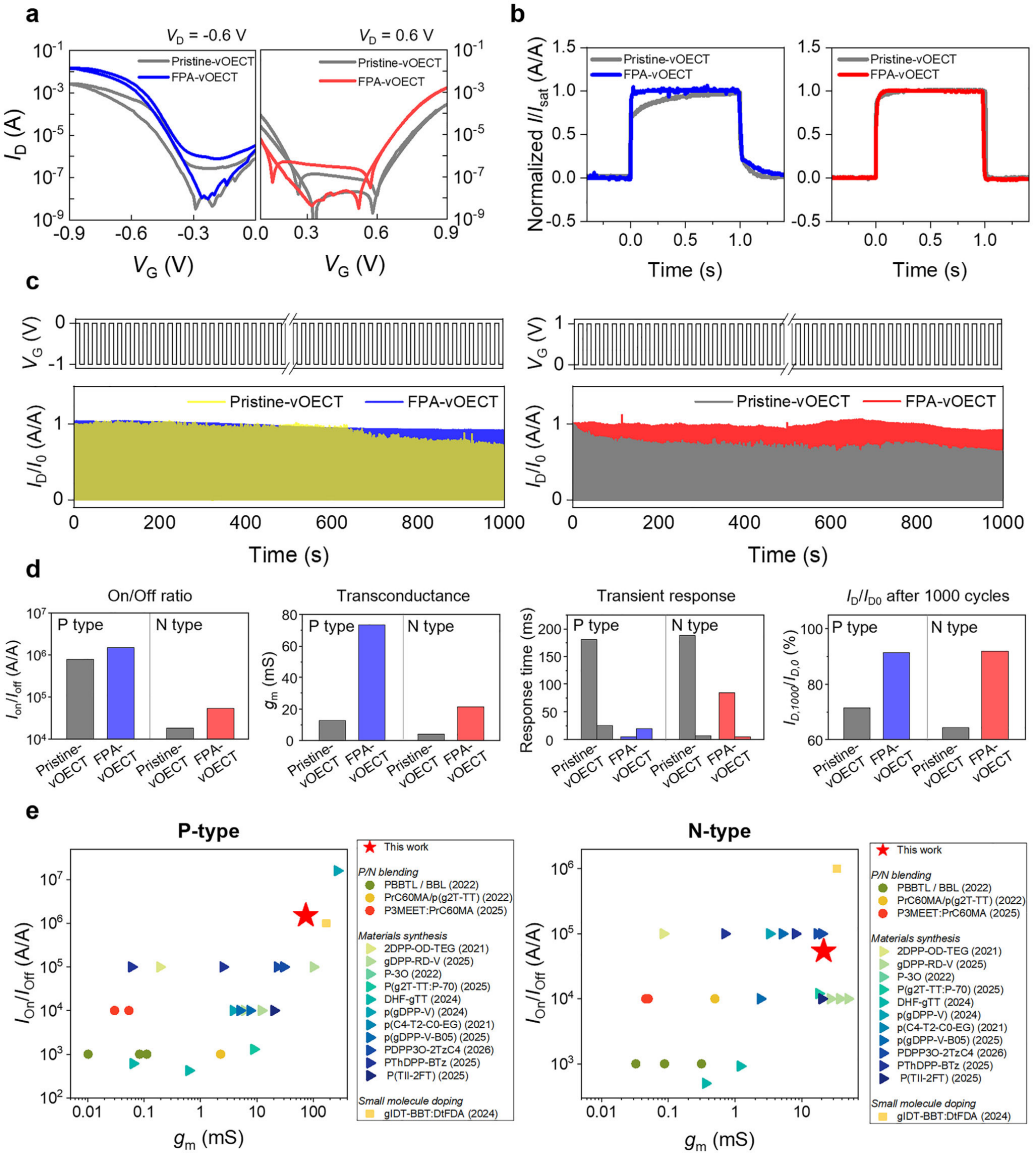
(a) Representative transfer characteristics of FPA‐vOECTs measured in 0.1 M of KCl(*aq*). Measurements were performed at *V*_D_ = −0.6 V with *V*_G_ swept from 0.3 to −0.9 V for p‐type operation, and at *V*_D_ = 0.6 V with *V*_G_ swept from −0.3 to 0.9 V for n‐type operation (sweep speed: 0.01 V s^−1^). (b) Transient responses under voltage pulses before and after pC6FA passivation, measured using 1 s square pulses of −1 and 1 V for p‐type and n‐type operation, respectively. (c) Pulse ‐stability measurements before and after pC6FA passivation in 0.1 M of KCl(*aq*). The pulse amplitudes were set to −1 and 1 V for p‐type and n‐type operation, respectively, while *V*_DD_ was fixed at −0.6 and 0.6 V for p‐type and n‐type operation, respectively. (d) Summary of extracted metrics including transconductance, on/off ratio, characteristic times, and current retention ratio (*I*_D,1000_/*I*_D,0_). (e) Performance comparison table summarizing the *g*_m_ and on/off ratio of the p‐type and n‐type operation modes of FPA‐vOECT, benchmarked against previously reported ambipolar OECTs.

The electrical characteristics of the FPA‐vOECT were compared with those of non‐passivated, pristine gDPP‐T‐based vOECT (pristine‐vOECT, afterward), which were subjected only to the identical thermal annealing condition to each other, to quantify the impact of the passivation. The representative transfer characteristics under both p‐type and n‐type operation are shown in Figure 3a. After the passivation, the turn‐on current increases from 2.7 to 14.4 mA in p‐type operation and from 0.025 to 1.5 mA in n‐type operation, accompanied by a simultaneous enhancement in the on/off current ratio from 0.8 × 10^6^ to 1.5 × 10^6^ and from 1.8 × 10^4^ to 5.4 × 10^4^ for p‐type and n‐type operation, respectively. In addition, the threshold voltage (*V*_th_) shifts toward lower absolute values for both operation modes, decreasing from −0.29 to −0.26 V in p‐type operation and from 0.58 to 0.52 V in n‐type operation. The operational hysteresis (*ΔV*) is also improved, from 0.055 to 0.021 V in p‐type operation and from 0.013 to 0.001 V in n‐type operation (*ΔV* is defined as *V*_reverse_ – *V*_forward_, where *V*_forward_ and *V*_reverse_ are extracted at *I*_D_ = 10^−5^ A). Consistent with these improvements in transfer characteristics, the *g*_m_, a key performance metric in OECTs that reflects signal amplification capability, shows a pronounced enhancement. Specifically, *g*_m_ increases from 12.7 (10.2 ± 6.6) to 73.7 (65.4 ± 25.6) mS in p‐type operation and from 4.1 (4.8 ± 4.5) to 21.4 (20.4 ± 9.6) mS in n‐type operation, highlighting the substantial amplification gain with efficiently suppressed hysteresis achieved through the fluoropolymer passivation. Statistical values were obtained from 16 devices, with a functional device yield of 100% for both pristine vOECTs and FPA‐vOECTs.

The transient response of the OECTs was further investigated by extracting the switching parameters (*τ*_on_ and *τ*_off_) (Figure 3b). Square voltage pulses with an amplitude of 1 V and a pulse width of 1 s were applied to the gate electrode, while biasing the drain voltage at −0.6 and +0.6 V for p‐type and n‐type operation, respectively, and the resulting transient drain current was recorded. The switching time of each case was extracted from the corresponding transient current profile by fitting the data with a double‐exponential function, as described in Equation (1) [49]. In this equation, the time constant *τ*_1_ is associated with the gDPP‐T OMIEC layer, whereas the time constant *τ*_2_ is associated with the pC6FA passivation layer. The faster time constant (*τ*_1_) was used to define the effective switching time, since the capacitance of the OMIEC channel is generally larger than that of the pC6FA passivation layer.

(1) $$I\;\left(t\right)=\left\{\begin{array}{c} I_{0}+A_{1}\left(1-e^{-\frac{t}{\tau_{1}}}\right)+A_{2}\left(1-e^{-\frac{t}{\tau_{2}}}\right)\;for\;\tau_{on}\\ I_{0}+B_{1}\left(e^{-\frac{t}{\tau_{1}}}\right)+B_{2}\left(e^{-\frac{t}{\tau_{2}}}\right)\;for\;\tau_{off} \end{array}\right.\;$$

As a result, the FPA‐vOECT exhibits markedly faster switching dynamics than the pristine devices in both turn‐on and turn‐off processes. Especially, *τ*_on_ decreases from 180.8 to 6.52 ms in p‐type operation and from 188.1 to 85.9 ms in n‐type operation. Similarly, *τ*_off_ is reduced from 25.7 to 20.9 ms for p‐type operation and from 7.7 to 6.4 ms for n‐type operation, indicating an overall acceleration of the transient response upon fluoropolymer passivation.

The electrochemical operational stability of the devices was also evaluated by repeated on/off switching cycles. As shown in Figure 3c, continuous square voltage pulses with an amplitude of 1 V and a pulse width of 1 s were repeatedly applied to the gate electrode for a total duration of 1000 s, while the drain current was recorded continuously. The pristine‐vOECT exhibits pronounced current fluctuation throughout the repeated cycling, both in n‐ and p‐operations, reflecting unstable operation even at the early stage of repeated switching. In contrast, the FPA‐vOECT maintains stable and reproducible current modulation over 1000 s of continuous operation with minimal signal drift. A comparison between the first and last switching cycles reveals that the current retention increases from 71.5% to 91.4% for p‐type operation and from 64.4% to 91.9% for n‐type operation, suggesting that fluoropolymer passivation substantially enhances the electrochemical stability of the channel under repeated ion‐doping processes and improves operational reproducibility in prolonged device operation. Figure 3d shows the overall performance gains in the on/off current ratio, transconductance, response time, and current retention after 1000 s of cyclic operation achieved through fluoropolymer passivation. A performance comparison table summarizing the transconductance and on/off current ratio of the FPA‐vOECT in both p‐type and n‐type operating modes is shown in Figure 3e [32, 33, 36, 50, 51, 52, 53, 54, 55, 56, 57, 58, 59, 60, 61]. In addition, the expanded table including operational stability and turn‐on/turn‐off response time information is provided in Table S2. Fluoropolymer passivation enhances the performance of gDPP‐T, delivering metrics that are competitive with those reported in previous studies.

To infer that the performance enhancement originates not only from the infiltration of polymer chains into the channel but also from the specific matrix‐domain nanostructure, we conducted a series of negative control tests involving variations in annealing and pC6FA deposition. As shown in Figure S9, the *g*_m_ of vOECTs fabricated under various process conditions was measured during p‐type operation. A comparison of the samples before and after annealing reveals an evident increase in *g*_m_ following the thermal treatment, which is in good agreement with previous reports in that annealing effectively improves the carrier mobility of the channel [19]. Notably, when the pC6FA deposition and the subsequent annealing process were performed together – a process that induces the matrix‐domain nanostructure – the *g*_m_ exhibited a dramatic surge. This improvement far exceeds a simple superposition of the individual effects from annealing‐induced mobility enhancement and passivation alone. These results strongly suggest the synergistic role of the unique nanostructure formed specifically after the pC6FA passivation followed by thermal annealing. In addition, we conducted the same passivation and annealing processes using other iCVD polymer layers with various functional groups. As shown in Figure S10, performance improvements in *g*_m_ were also observed for poly(1‐vinyl imidazole) (pVIDZ), poly(4‐dimethylamino methylstyrene) (pDMAMS), poly(di(ethylene glycol) ethyl ether acrylate) (pDEGEEA), and poly(isononyl acrylate) (pINA). We could observe general improvements from the devices passivated with other iCVD polymer coatings, the enhancement achieved with pC6FA passivation was exceptionally superior, confirming its unique effectiveness in optimizing device performance.

### 2.3 Electrochemical Redox and Optoelectronic Behaviors

The electrical characteristics of the FPA‐vOECT are significantly improved compared with those of the pristine‐vOECT and negative controls using passivation with various polymers. These results suggest that the matrix‐domain nanostructure formed after pC6FA passivation plays a critical role in modulating the electrochemical doping behavior of the channel. To elucidate the mechanistic origin of the improvements enabled by the fluorine‐rich matrix‐domain nanostructure, the electrochemical behavior of the gDPP‐T layer was systematically investigated.

**FIGURE 4:**
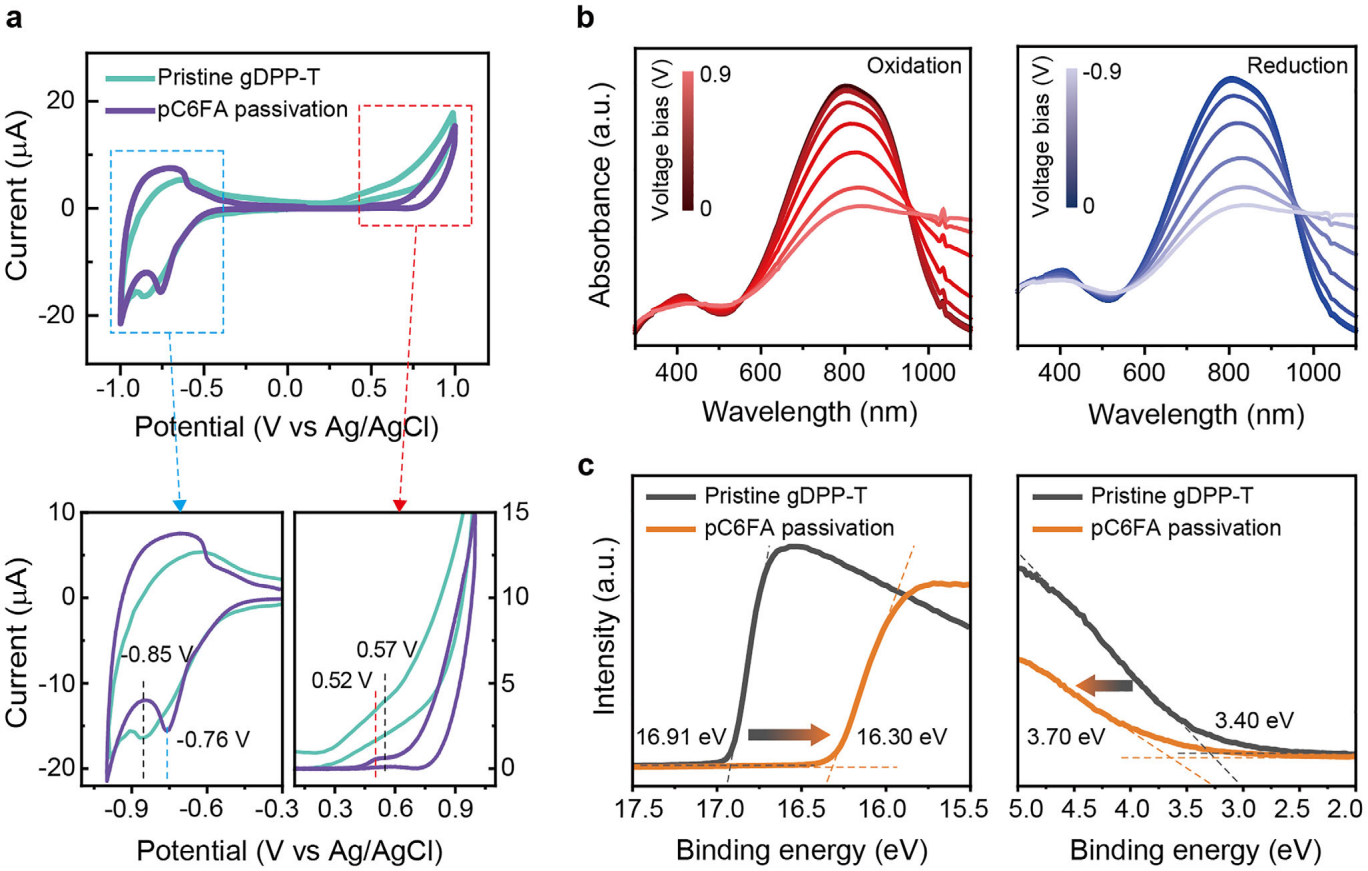
(a) CV of pristine gDPP‐T (sky blue) and pC6FA‐passivated gDPP‐T (purple). The boxed regions highlight the oxidation (red) and reduction (blue) sweeps, shown as magnified traces below. (b) Electrochemical absorption spectra of pC6FA‐passivated gDPP‐T in 0.1 M KCl aqueous electrolyte under oxidation (red) and reduction (blue) at various bias against the Ag/AgCl reference electrode. (c) UPS spectra of pristine gDPP‐T (black) and pC6FA‐passivated gDPP‐T (orange), showing the SECO (left) and HOMO onset (right) regions.

Cyclic voltammetry (CV) was first employed to examine the redox characteristics of the channel layer (Figure 4a). Both pristine and pC6FA‐passivated gDPP‐T films exhibit well‐defined oxidation and reduction peaks, confirming that the intrinsic ambipolar redox activity of the polymer is preserved after fluoropolymer passivation. After passivation, the oxidation onset shifts from 0.57 to 0.52 V, while the reduction onset shifts from −0.85 to −0.76 V (vs Ag/AgCl). The simultaneous shift of both redox processes toward lower absolute potentials indicates improved ion accessibility in the passivated films, enabling electrochemical charging of the channel at lower bias. This shift is consistent with the observed decrease in *V*_th_ in both p‐type and n‐type OECT operation (Figure 3a), suggesting that the passivated channel requires a smaller gate bias to access comparable doping states. In addition to the redox onset shift, electrochemical cycling stability was further evaluated by analyzing the CV response over the full potential window (Figure S11). In the pristine gDPP‐T film, repeated CV cycling gradually decreases the absolute current value of the reduction onset potential, leading to a progressive reduction in the overall enclosed area. By contrast, the pC6FA‐passivated gDPP‐T film shows negligible changes in the reduction of onset current upon repeated scans, indicating a stable electrochemical response. Because the enclosed area reflects the amount of charge transferred during the redox process, the shrinking area in the pristine film suggests that the charge injected into and extracted from the channel layer diminishes over successive cycles. In comparison, the passivated film maintains an almost constant charge transfer even under repeated redox cycling, consistent with a more reproducible redox reaction [17, 62]. This stabilized electrochemical behavior aligns with the reduced operational hysteresis (Δ*V*) and enhanced current reproducibility observed in the OECT characteristics (Figure 3a–c), indicating that fluoropolymer passivation promotes a more reliable electrochemical gating process.

To directly probe electrochemical doping states, in situ electrochemical ultraviolet‐visible (UV–vis) absorption spectroscopy was performed in 0.1 M KCl aqueous electrolyte (Figure 4b). Under both oxidative and reductive potentials, the passivated film exhibits characteristic spectral signatures of doping, including an increase in long‐wavelength absorption (> 1000 nm), accompanied by suppression of the π–π* transition (∼400 nm) and bleaching of the intermolecular charge‐transfer (ICT) band (650–1000 nm). These spectral features are widely recognized as signatures of polaron/bipolaron formation in conjugated polymers [20, 32, 63]. Compared with the pristine film (Figure S12a,c), the ICT band bleaching is more pronounced after passivation, implying more effective formation and stabilization of polaronic/bipolaronic species in the passivated film both oxidation and reduction (Figure S12b,d, respectively). The well‐defined and bias‐responsive optical modulation is consistent with the increased drain current (*I*_on_) and enhanced transconductance (*g*_m_) observed in the FPA‐vOECT (Figure 3a,d).

To further clarify whether the fluoropolymer passivation modifies the interfacial electronic environment relevant to electrochemical gating, ultraviolet photoelectron spectroscopy (UPS) was performed on pristine and pC6FA‐passivated gDPP‐T films. Figure 4c illustrates the UPS results. The secondary electron cutoff (SECO) and the HOMO onset were analyzed because they provide direct information on the surface work function and the valence electronic edge position. By linear extrapolation of the SECO edge, the cutoff shifts from 16.91 to 16.30 eV after passivation, indicating an increase in work function. By linear extrapolation of the valence‐band edge, the HOMO onset shifts from 3.40 to 3.70 eV relative to the Fermi level [64]. These concurrent shifts indicate that the interfacial electronic energetics are altered after pC6FA passivation. Such vacuum‐level realignment is consistent with a modified surface electronic environment, which may be related to the improved bias efficiency and reduced hysteresis observed in the FPA‐vOECT devices.

Based on all the observations above, pC6FA passivation facilitates electrochemical doping at lower bias while stabilizing the redox response during repeated cycling. Along with the observed vacuum‐level realignment, these results suggest that enhanced performance and operational stability arise from both improved ion‐accessible electrochemical gating and a modified interfacial electronic environment.

### 2.4 Application to Electrocardiogram (ECG) Signal Processing

To explore the system‐level applicability of the developed high‐performance and operationally stable FPA‐vOECT, we employed the device as a fundamental building block in a neuromorphic encoding architecture. In particular, to evaluate its suitability for neuromorphic health monitoring systems, we implemented a circuit that encodes human biosignals, with a focus on electrocardiogram (ECG) signals, and subsequently utilized the encoded outputs as inputs to a neural network for ECG signal classification. Through this approach, we assess not only the device‐level electrical performance but also its functional relevance in biosignal processing and intelligent diagnostic applications. ECG signals capture the electrical activity associated with cardiac contraction and relaxation to provide critical information for arrhythmia diagnosis. Recently, several studies have proposed AI‐based approaches that diagnose cardiac states and related health problems by training AI models on ECG waveforms. For effective utilization of AI, physiological signals must first be represented in an AI‐adaptable data form. In conventional signal processing schemes, ECG signals are converted into digital signals using analog‐to‐digital converters (ADCs), stored in memory units, and subsequently processed by digital computing architectures. However, this processing paradigm inevitably incurs significant overhead in terms of energy consumption and data latency, rendering it inefficient and impractical for continuous, real‐time health monitoring systems.

In this study, we propose, for the first time, an event‐based organic bipolar spiking (e‐ORBIS) neuromorphic encoder using the FPA‐vOECT as an essential building block. The passivated FPA‐vOECT exhibits improved operational stability, faster response, and enhanced switching characteristics, making it suitable for repeated and reliable signal‐encoding operation in circuit‐level applications. The e‐ORBIS encoder is designed for energy‐efficient operation by combining event‐driven spike representation – generating output spikes only in response to significant input changes while remaining silent during steady‐state intervals – with a low‐voltage operating system enabled by the FPA‐vOECT. In addition, owing to the intrinsic ambipolarity of the FPA‐vOECT, the e‐ORBIS circuit can encode both upward and downward variations of the input signal through a structurally simple configuration in which only the inverter voltage polarity is reversed. By producing two distinct outputs from a single input waveform, the encoder preserves temporal information from both p‐type and n‐type modes, thereby providing far more enriched representations for downstream artificial neural network (ANN) processing.

**FIGURE 5:**
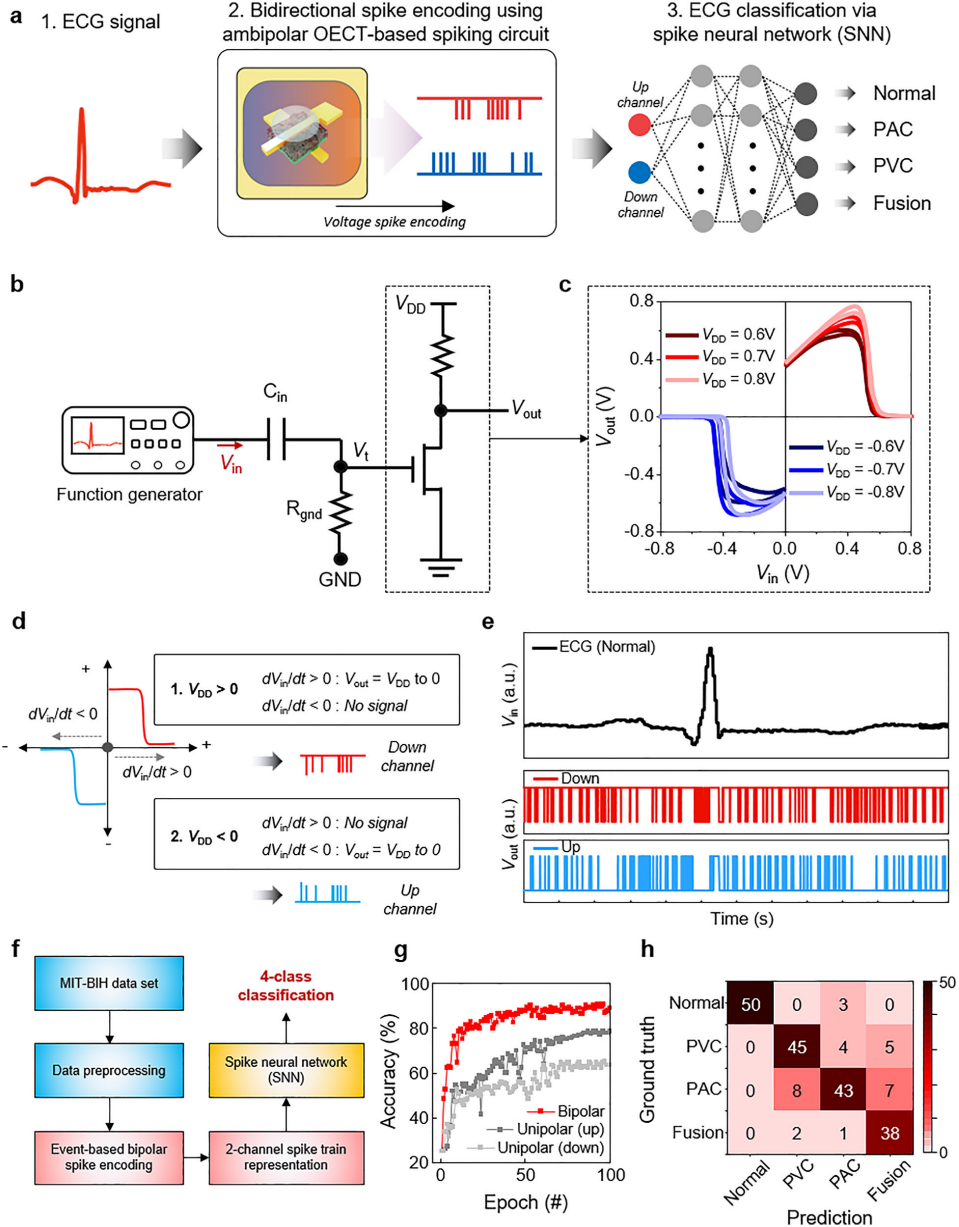
(a) Schematic overview of physiological signal processing using an ambipolar OECT‐based bipolar neural encoding system integrated with a spiking neural network. (b) Circuit diagram of the event‐based bipolar spike encoder circuit implemented with an iCVD‐passivated ambipolar OECT. (c) Voltage transfer characteristics (VTCs) of the bipolar inverter with a series load resistor measured under varying *V*_DD_. (d) Encoding mechanism and (e) UP and DOWN spike trains obtained through post‐processing of experimentally encoded normal‐class ECG signals. (f) Processing pipeline for MIT‐BIH arrhythmia classification employing the proposed bipolar spiking encoder. (g,h) Classification performance evaluated by overall accuracy and the corresponding confusion matrix.

Figure 5a shows the overall ECG signal encoding and AI‐based classification process. When an analog ECG signal is injected into the e‐ORBIS, positive and negative temporal variations of the input signal are selectively encoded into oppositely directed spike events. The resulting bipolar spike trains provide an event‐driven representation of the ECG signal that can be directly interfaced with spiking neural networks (SNNs), enabling subsequent ECG classification without additional analog‐to‐digital conversion. Figure 5b shows the experimental schematic of the proposed e‐ORBIS system demonstration. The system consists of a series capacitor and an FPA‐vOECT/load‐resistor inverter. The ECG input generated by a function generator is applied through the series capacitor and converted into event‐driven voltage variations according to Equation (2).

(2) $$\Delta V_{C_{\mathrm{in}}}\left(t\right)\propto C\frac{dV_{\mathrm{in}}\left(t\right)}{\mathrm{dt}}\;$$

The resulting node potential serves as the input voltage of the FPA‐vOECT (*V*_t_), inducing transient changes in the inverter output (*V*_out_) and generating spike signals.

The electrical characteristics of the bipolar inverter, composed of the FPA‐vOECT and a load resistor, were characterized. Figure 5c shows the voltage transfer characteristics (VTCs) of the bipolar inverter, where clear signal inversion is achieved in both the p‐type (−0.8 to 0 V) and the n‐type operation region (0 to 0.8 V). Increasing *V*_DD_ from 0.6 to 0.8 V leads to a corresponding increase in the output voltage, confirming the proper inverter operation. Signal inversion occurs around −0.4 and 0.6 V with peak voltage gains of 13.3 V/V and 13.8 V/V in the p‐type and n‐type regions, respectively (Figure S13). Besides the static transfer characteristics, the switching behavior of the FPA‐vOECT‐based bipolar inverter was evaluated. As shown in Figure S14, stable input/output voltage characteristics are maintained over 800 s of continuous operation. From these measurements, the response times were extracted, yielding *τ*_on_ = 877 ms and *τ*_off_ = 84 ms for p‐type operation, and *τ*_on_ = 234 ms and *τ*_off_ = 122 ms for n‐type operation, demonstrating reliable dynamic operation in both regimes.

The working principle of the e‐ORBIS is illustrated in Figure 5d. When the supply voltage is set to *V*_DD_ > 0, a positive change in the input voltage (*dV*_in_/*dt* > 0) induces a positive transient voltage at node, which increases *V*_OECT_ of the ambipolar OECT inverter. Once *V*_OECT_ exceeds the inverter threshold, the *V*_out_ switches from *V*_DD_ to 0, generating a spike. In contrast, a negative change in the input voltage (*dV*_in_/*dt* < 0) produces an opposite transient response that does not trigger inverter switching, and no spike is generated. When the supply voltage polarity is reversed (*V*_DD_ < 0), the encoding behavior is inverted accordingly. In this case, a negative temporal change in the input voltage decreases *V*_OECT_, and a spike is generated when the inverter threshold is crossed, whereas a positive temporal change does not induce spike generation. This simple encoding scheme exploits two key advantages of the FPA‐vOECT. First, owing to its intrinsic ambipolar characteristics, signal inversion is achieved for both positive and negative supply polarities, enabling event‐based encoding of both increasing and decreasing voltage dynamics without additional circuit elements. Second, the low operating voltage of the OECT facilitates inverter switching under small voltage variations, allowing event‐driven voltage changes at the input node to modulate the output through a simple inverter configuration.

Figure 5e presents the UP and DOWN spike trains obtained through post‐processing of experimentally encoded normal‐class ECG signals. When a representative ECG‐derived input was applied as *V*_in_ to the e‐ORBIS, spike events were generated, and the signal amplitude at each spike occurrence was digitized to construct the spike trains. As a result, spike outputs are generated selectively during periods where *V*_in_ exhibits temporal variations, confirming event‐driven spike encoding behavior. Furthermore, by adjusting *V*_DD_, selective spiking responses to the polarity of Δ*V* are observed, demonstrating polarity‐dependent bipolar spike encoding. Then, to evaluate whether the proposed event‐based spike encoder provides appropriate input representations for neural network training, an ECG arrhythmia classification simulation was conducted. The simulation followed the processing pipeline illustrated in Figure 5f. Raw ECG data were obtained from the MIT‐BIH arrhythmia dataset, from which samples corresponding to four classes – normal, premature ventricular contraction (PVC), premature atrial contraction (PAC), and fusion – were randomly selected. A total of 1000 samples were used, among which 800 samples were allocated for training, and the remaining 200 samples were used for testing. Based on this experimentally demonstrated encoding behavior, an ECG arrhythmia classification simulation was conducted using a corresponding encoding model to evaluate the utility of the bipolar spiking encoder for neural processing. The extracted raw ECG signals underwent simple preprocessing and were then processed using a device‐informed encoding simulation model constructed from the experimental data of the fabricated FPA‐vOECT. Through this process, the ECG signals were converted into up‐ and down‐channel bipolar spike trains, which were subsequently fed into the SNN for arrhythmia classification. Further details are provided in the Experimental section.

Figure 5g,h summarize the classification performance of the proposed bipolar event‐based encoding system, showing the classification accuracy as a function of training epochs and the corresponding confusion matrix, respectively. When the up‐ and down‐channel spike trains generated using this bipolar encoding model were used as inputs for neural network training, the system achieved a peak classification accuracy of 90.5% at 100 training epochs. Notably, when the network was trained using unipolar spike encoding alone, the classification accuracy dropped significantly to 78.6% and 63.5% for the up‐channel and down‐channel inputs, respectively. This substantial performance gap demonstrates that bipolar spike encoding, realized through the ambipolar switching behavior of the FPA‐vOECT, delivers more informative and complementary temporal features than unipolar encoding. Unlike conventional CMOS implementations, in which separate p‐type and n‐type devices are required, and simple polarity reversal cannot realize both p‐type and n‐type derived encoding within a single inverter structure, the ambipolar OECT‐based inverter enables bipolar signal encoding merely by reversing the supply polarity, without modifying the circuit topology. Therefore, beyond comparable classification capability, the proposed e‐ORBIS encoder offers a structurally simplified architecture while simultaneously enhancing encoding richness, highlighting a distinctive functional advantage of ambipolar devices for neuromorphic biosignal processing.

## 3 Conclusions

In this work, we introduce, for the first time, an iCVD‐based fluoropolymer passivation strategy in which pC6FA is directly deposited onto gDPP‐T ambipolar OMIEC as an effective route to enhance OECT performance. This single post‐treatment simultaneously improves both hole‐ and electron‐dominated conduction, representing the first demonstration of concurrent performance enhancement in p‐type and n‐type ambipolar OECT operation through a unified process. Electrical and electrochemical analyses reveal that the improvement originates from the formation of a fluorine‐rich domain–conductive matrix nanostructure established between pC6FA and gDPP‐T after passivation and thermal annealing, which modulates ion diffusion pathways and enables more efficient doping/dedoping dynamics. As a result, the FPA‐vOECT exhibits remarkable performance enhancements in transconductance (from 12.7 to 73.7 mS in p‐type and from 4.1 to 21.4 mS in n‐type operation), transient response (*τ*_on_ reduced from 180.8 to 6.5 ms in p‐type and from 188.1 to 85.9 ms in n‐type), and operational stability (current retention of 91.4% and 91.9% for p‐type and n‐type modes, respectively, after 1000 s of repeated switching). Finally, we demonstrate an event‐based organic bipolar spiking (e‐ORBIS) encoder for biosignal processing using the FPA‐vOECT as a key component. Based on a structurally simple circuit configuration, ECG signals were encoded through ambipolar‐enabled bipolar operation, and subsequent neural network simulations achieved a peak classification accuracy of 90.5%, substantially outperforming unipolar encoding schemes (78.6% and 63.5%), thereby confirming that ambipolar‐enabled bipolar spike encoding provides richer temporal information within a simplified inverter architecture.

## 4 Experimental

### 4.1 Materials

The monomers used for the passivation layers, including 2‐(perfluorohexyl)ethyl acrylate (C6FA), 1‐vinylimidazole (VIDZ), 4‐(dimethylamino)methylstyrene (DMAMS), di(ethylene glycol)ethyl ether acrylate (DEGEEA), and isononyl acrylate (INA), as well as tert‐butyl peroxide (TBPO) used as the initiator, were purchased from Sigma–Aldrich. All materials were used in the iCVD process without further purification. The gDPP‐T polymer used as the OMIEC layer was purchased from Derthon.

### 4.2 OMIEC Solution Preparation

The semiconductor solution was prepared by dissolving 40 mg of gDPP‐T in 2 mL of chlorobenzene. The solution was stirred for 24 h to obtain a concentration of 20 mg mL^−1^.

### 4.3 FPA‐vOECT Fabrication

Lime‐free glass substrates were used for device fabrication. The substrates were cleaned by ultrasonication in deionized water (DIW), acetone, and isopropyl alcohol (IPA) sequentially for 10 min each. For the bottom electrode of the vOECT, a 100 nm of Au layer was deposited by thermal evaporation at a rate of approximately 1 Å s^−1^ using a shadow mask with a channel width of 100 µm. Subsequently, the OMIEC solution was spin‐coated at 3000 rpm for 30 s, followed by drying in an N_2_‐filled glove box to remove residual solvent, forming an active channel layer with a thickness of approximately 150 nm. Afterward, the iCVD process was carried out to deposit the pC6FA passivation layer, with a thickness of approximately 30 nm, and the detailed process conditions for pC6FA deposition are described in Figure S3a. The top electrode was then formed by thermal evaporation of a 150 nm of Au layer at a rate of approximately 1 Å s^−1^ using a shadow mask with a width of 100 µm. After completion of the layer integration process, thermal annealing was performed at 150°C for 30 min. For the electrolyte and gate electrode, a 0.1 M aqueous KCl solution (10 µL) was directly drop‐cast onto the device, and an Ag/AgCl‐coated gold probe tip was used as the gate electrode.

### 4.4 Electrical Characterization

Electrical characterization of the FPA‐vOECTs, inverters, and event‐based spiking systems was carried out using an Agilent B1500A semiconductor parameter analyzer. A 0.1 M KCl aqueous solution was used as the electrolyte. The voltage sweep rate was fixed at 0.1 V s^−1^ for all measurements. For the measurement of the device response time and operational stability, pulse operation was performed using a pulse generator. Specifically, a function generator (AFG 3021, Tektronix) was used to generate voltage pulses, and an oscilloscope (TDS 2024B, Tektronix) was employed to monitor the device response, enabling accurate capture of the fast transient characteristics. All measurements were performed under ambient conditions.

To optimize the pC6FA thickness, films with different thicknesses were deposited, and the resulting transconductance values were extracted from the transfer curves using Equation (3):

(3) $$g_{m}=\frac{\partial I_{D}}{\partial V_{G}}\;$$

While the n‐type transconductance showed only a weak dependence on thickness, the p‐type transconductance reached a maximum at 30 nm (Figure S2). Therefore, a pC6FA thickness of 30 nm was selected as the optimal condition for subsequent fabrication and analysis of FPA‐vOECTs.

### 4.5 Material Characterization

Time‐of‐flight secondary ion mass spectrometry (ToF‐SIMS) was performed using a TOF‐SIMS 5 instrument (ION‐TOF GmbH). Fourier‐transform infrared (FT‐IR) spectra were recorded using an ALPHA FT‐IR spectrometer (Bruker Optics). Cyclic voltammetry (CV) was carried out using a CHI600 electrochemical workstation (CH Instruments). Ultraviolet photoelectron spectroscopy (UPS) spectra were collected using an AXIS Supra system (Kratos). UV–vis spectra were obtained using a UV‐1260 spectrophotometer (Agilent).

### 4.6 Realization of e‐ORBIS

The e‐ORBIS was constructed by connecting the FPA‐vOECT with a 100 kΩ load resistor to form an inverter configuration. A ground resistor (*R*_gnd_) of 1 MΩ and an input series capacitor (*C*_in_) of 10 pF were connected to the input node. For the ECG encoding experiment, one representative normal ECG waveform was randomly selected from the MIT‐BIH arrhythmia dataset and used to prepare the input waveform. The waveform was generated using a function generator. To obtain sufficient transient voltage variations through the series capacitor, the signal amplitude was adjusted during waveform generation, and the ECG waveform was applied in a step‐pulsed form. This input condition was used for the proof‐of‐concept demonstration of spike generation using the FPA‐vOECT‐based inverter.

### 4.7 Neural Network Simulation for Arrhythmia Detection

The spike‐encoded ECG signals were classified using a spiking neural network (SNN). Specifically, the proposed SNN received two‐channel spike trains corresponding to the up‐ and down‐spike outputs generated by the bipolar encoder, where each ECG sample consisted of a temporal sequence with 200 time steps. The SNN consisted of two parallel hidden branches of leaky integrate‐and‐fire (LIF) neurons with different membrane time constants (*τ*_fast_ = 6 and *τ*_slow_ = 20), enabling multi‐timescale temporal processing. The parallel LIF branches were designed to capture short‐ and long‐timescale temporal dynamics of the ECG signal. The spike outputs from each branch were further divided into early‐, middle‐, and late‐temporal windows and pooled independently, after which all temporal features were concatenated and fed into a fully connected synaptic readout layer for four‐class classification (normal, PVC, PAC, and fusion). The network was trained using cross‐entropy loss and optimized via backpropagation with surrogate gradients. The synaptic weights, including the trainable channel‐combination weights for the up‐ and down‐spike streams, were randomly initialized using Xavier initialization and optimized using the Adam optimizer for 100 epochs with a batch size of 64. In addition, temporal jitter augmentation of ±3‐time steps was applied during training to improve robustness against temporal variations in ECG waveforms. The MIT‐BIH arrhythmia dataset used in this work consisted of 1000 ECG samples, among which 800 samples were used for training, and the remaining 200 samples were used for testing.

## Author Contributions

J.Y. and B.L. contributed equally to this work. J.Y., B.L., Y.K., and S.G.I. conceived the research idea and designed the experiments. H.L., M.K., C.C., and J.A.L. assisted with device fabrication and characterization. All authors discussed and analyzed the data. J.Y., B.L., Y.K., and S.G.I. wrote the manuscript. All authors reviewed the manuscript. Y.K. and S.G.I. contributed equally as corresponding authors.

## Conflicts of Interest

The authors declare no conflicts of interest.

## Supporting information




**Supporting File**: advs77167‐sup‐0001‐SuppMat.docx.

## Data Availability

The data that support the findings of this study are available from the corresponding author upon reasonable request.
