# Supplementary material for: Damage‐Free Passivation of Ambipolar OECTs with Fluoropolymer Film for Enhanced Performance and Stability Toward Reliable Biosignal Processing
Source: Adv Sci (Weinh). 2026 Sep 2:e77167. Online ahead of print. doi: 10.1002/advs.77167 (PMC13535796; doi:10.1002/advs.77167)
Supplement: Supplementary file 1 [file ADVS-9999-e77167-s001.docx]

Supporting Information

**Damage-Free Passivation of Ambipolar OECTs with Fluoropolymer Film for Enhanced Performance and Stability toward Reliable Biosignal Processing**

Junyeong Yang^1†^, Bohyun Lee^1†^, Hanna Lee^2,3^, Mingyu Kim^4^, Changsoon Choi^5,6^, Jung Ah Lim^2,7^, Youson Kim^5^*, and Sung Gap Im ^1,4^*

^1^*Department of Chemical and Biomolecular Engineering, Korea Advanced Institute of Science and Technology (KAIST), Yuseong-gu, Korea.*

^2^*Electronic and Hybrid Materials Research Center, Korea Institute of Science and Technology (KIST), Seoul 02792, Republic of Korea.*

^3^*GIST InnoCORE AI-Nano Convergence Institute for Early Detection of Neurodegenerative Diseases, Gwangju Institute of Science and Technology, Gwangju 61005, Republic of Korea*

^4^*KAIST Graduate School of Semiconductor Technology, KAIST, Yuseong-gu, Korea.*

^5^*Center for Quantum Technology, Post-silicon Semiconductor Institute, Korea Institute of Science and Technology (KIST), Seoul 02792, Republic of Korea.*

^6^*Department of Electrical and Computer Engineering, Sungkyunkwan University (SKKU), Suwon 16419, Republic of Korea.*

^7^*Division of Nano and Information Technology KIST School, University of Science and Technology of Korea (UST), Seoul 02792, Republic of Korea.*

^†^*J. Yang and B. Lee contributed equally to this work.*

*To whom correspondences should be addressed.

*E-mail: sgim@kaist.ac.kr (S.G. Im) and kimyouson@kist.re.kr (Y. Kim)*

**Table S1.** Summary of reported alternative strategies for OMIEC development beyond chemical synthesis.

| Journal / Year | Types of semiconductors | Strategy | Device structure | *g*_m_ (mS) | *I*_ON_/*I*_OFF_ | μC* (F cm^-1^ V^-1^ s^-1^) | Stability (cycles) | τ_ON_ (ms) | τ_OFF_ (ms) |
| --- | --- | --- | --- | --- | --- | --- | --- | --- | --- |
| Nature, 2023 [1] | gDPP-g2T:Cin-Cell | Blending | Vertical | 203.5 | ~10^5^ | N/A | 50000 | 425 | 85 |
| Nature, 2023 [1] | Homo-gDPP:Cin-Cell | Blending | Vertical | 101.2 | ~10^7^ | N/A | 50000 | 366 | 45 |
| Nat Electron, 2025 [2] | BBL/PEI | Bilayer | Vertical | 13.7 | 3×10^6^ | N/A | 1000000 | 27 | N/A |
| Nat Electron, 2025 [3] | p(g3T2):C_60_F_48_ | Degassing +Blending | Planar | 25 | 1.4×10^6^ | 680 | 4600 | N/A | N/A |
| J Appl Phys, 2021 [4] | P3HT/PMMA | Bilayer | Planar | N/A | 10^4^ | N/A | N/A | N/A | N/A |
| ACS Mater Au. 2023 [5] | P75:TBAClO_4_ | Blending | Planar | 5.1×10^-3^ | 10^4^ | 0.24 | N/A | 7.4×10^3^ | 1.6×10^4^ |
| npj Flex Electron, 2025 [6] | P3MEET:PTES | Blending | Planar | 45 | ~10^3^ | 192.9 | ~160 | 2.9×10^6^ | N/A |
| Adv Mater, 2024 [7] | p(N-T):PS10K | Blending | Planar | 0.43 | N/A | 13.4 | ~2000 | 2.9×10^3^ | 1.0×10^3^ |
| Angew Chem Int Ed, 2024 [8] | p(g2T-T):DtFDA | Blending +UV | Vertical | 68.6 | 6.7×10^5^ | N/A | 100000 | 9.3×10^2^ | 1.8×10^2^ |
| Angew Chem Int Ed, 2024 [8] | Homo-gDPP:DtFDA | Blending +UV | Vertical | 81.6 | 3.9×10^6^ | N/A | 100000 | 9.2×10^2^ | 2.9×10^2^ |

**Table S2.** Performance comparison of ambipolar organic electrochemical transistors (OECTs) based on various enhancement strategies.

| **Journal, Year** | **Types of semiconductors** | **Strategy** | **Device structure** | **p-type** | | | | | | **n-type** | | | | | |
| --- | --- | --- | --- | --- | --- | --- | --- | --- | --- | --- | --- | --- | --- | --- | --- |
|  |  |  |  | ***g*_m_ (mS)** | ***I*_on_/*I*_off_** | **Stability (cycles)** | **τ_on_ (ms)** | **τ_off_ (ms)** | ***g*_m_ (mS)** | | ***I*_on_/*I*_off_** | **Stability (cycles)** | **τ_on_ (ms)** | **τ_off_ (ms)** |  |
| Adv. Mater., 2022 [9] | PB[9]BTL/BBL | P/N blending | Planar | 0.084 | < 10^3^ | 5,000 | 3.05 | 1.95 | 0.088 | | ~10^3^ | 5,000 | 1.72 | 0.38 |  |
| Nat. Commun., 2022 [10] | P(g2T-TT) /PrC_60_MA | P/N blending | Planar | 0.96 | < 10^3^ | 100 | 466 | 5.8 | 0.60 | | ~10^4^ | 100 | 20 | 5.6 |  |
| ACS Appl. Mater. Interfaces, 2025 [11] | P3MEEMT /PrC_60_MA | P/N blending | Planar | 0.0675 | ~10^3^ | N/A | N/A | N/A | 0.0625 | | ~10^4^ | N/A | N/A | N/A |  |
| ACS Appl. Mater. Interfaces, 2025 [11] | P3MEEMT /PTEG-1 | P/N blending | Planar | 0.0375 | < 10^3^ | N/A | N/A | N/A | 0.0575 | | ~10^4^ | N/A | N/A | N/A |  |
| Adv. Funct. Mater., 2021 [12] | 2DPP-OD-TEG | Chemical synthesis | Planar | 0.19 | ~10^5^ | 166 | ~1800 | < 500 | 0.084 | | > 10^5^ | 166 | < 500 | < 500 |  |
| Adv. Mater., 2025 [13] | 3gDPP-RD-V (KCl solution) | Chemical synthesis | Planar | 7.5 | ~10^4^ | N/A | 502.3 | 19.7 | 26.8 | | ~10^4^ | N/A | 375 | 5.0 |  |
| Adv. Mater., 2025 [13] | 2gDPP-RD-V (KCl solution) | Chemical synthesis | Planar | 11.0 | ~10^4^ | N/A | 2900 | 25.3 | 37.1 | | ~10^4^ | N/A | 502.8 | 12.2 |  |
| Adv. Mater., 2025 [13] | 2gDPP-RD-V (KPF_6_ solution) | Chemical synthesis | Planar | 93.2 | ~10^5^ | N/A | 1470 | 32.8 | 49.1 | | ~10^4^ | N/A | 1240 | 6.1 |  |
| Adv. Mater., 2022 [14] | P-3O | Chemical synthesis | Planar | N/A | ~10^4^ | 21,600 | N/A | N/A | N/A | | ~10^4^ | 21,600 | N/A | N/A |  |
| ACS Materials Lett., 2026 [15] | P(g2T-TT)/P-7O | Chemical synthesis & P/N blending | Planar | 8.8 | 1.3×10^3^ | > 10,000 | 16.49 | Fast (N/A) | 14.48 | | 1.2×10^4^ | > 10,000 | 1.69 | 0.89 |  |
| Adv. Funct. Mater., 2025 [16] | DHF-gTT | Chemical synthesis | Planar | 0.624 | 4.2×10^2^ | N/A | 38.75 | 5.87 | 1.00 | | 9.2×10^2^ | N/A | 5.87 | 1.57 |  |
| Adv. Funct. Mater., 2025 [16] | DH-gTT | Chemical synthesis | Planar | 0.064 | 6.1×10^2^ | N/A | 41.32 | 6.07 | 0.364 | | 5.0×10^2^ | N/A | 5.32 | 1.24 |  |
| Sci. Adv., 2024 [17] | P(gDPP-V) | Chemical synthesis | Planar | 3.8 | ~10^4^ | >10,000 | 63 | 24 | 3.3 | | ~10^5^ | >10,000 | 41 | 22 |  |
| Sci. Adv., 2024 [17] | P(gDPP-V) | Chemical synthesis | Vertical | 267.5 | 1.6×10^7^ | 10,000 | < 2 | N/A | 263.2 | | 5.9×10^7^ | 10,000 | < 2 | N/A |  |
| Sci. Adv., 2021 [18] | P(C4-T2-C0-EG) | Side chain engineering | Planar | 0.00819 | ~10^4^ | N/A | N/A | N/A | 0.00921 | | ~10^3^ | N/A | N/A | N/A |  |
| Adv. Mater., 2026 [19] | P(gDPP-V-B05) | Side chain engineering | Planar | 7.5 | ~10^4^ | 30 | 217.8 | 83.8 | 5.1 | | ~10^5^ | 30 | 348.5 | 58.1 |  |
| Adv. Mater., 2026 [19] | P(gDPP-V-B20) | Side chain engineering | Planar | 5.0 | ~10^4^ | 30 | 255.5 | 104.8 | 2.4 | | ~10^4^ | 30 | 402.1 | 116.5 |  |
| Adv. Sci., 2026 [20] | PDPP3O-2TzC4 | Chemical synthesis | Planar | 28.33 | > 10^5^ | < 1,500 | 3.75 | 0.85 | 20.91 | | > 10^5^ | < 100 | 3.81 | 1.01 |  |
| Adv. Sci., 2026 [20] | PDPP5O-2TzC4 | Chemical synthesis | Planar | 22.68 | > 10^6^ | 1,500 | 1.82 | 0.46 | 15.70 | | > 10^6^ | < 100 | 1.85 | 0.85 |  |
| J. Mater. Chem. C, 2025 [21] | PThDPP-BTz | Chemical synthesis | Planar | 0.06 | ~10^4^ | N/A | 102.6 | 41.1 | 0.7 | | > 10^4^ | N/A | 23.7 | 7.4 |  |
| J. Mater. Chem. C, 2025 [21] | PThDPP-BBTz | Chemical synthesis | Planar | 2.5 | ~10^2^ | N/A | 133.2 | 11.1 | 8.0 | | > 10^2^ | N/A | 113.1 | 19.4 |  |
| Nat. Commun., 2025 [22] | P(TII-2FT) | Chemical synthesis | Vertical | N/A | ~10^5^ | 1,000 | 1.32 | 0.94 | N/A | | ~10^5^ | 1,000 | 4.07 | 1.53 |  |
| Aggregate, 2024 [23] | gIDT-BBT/DtFDA | Chemical synthesis & crosslinker blend | Vertical | 155.05 | > 10^6^ | 2,000 | 170.33 | 9.97 | 27.28 | | > 10^6^ | 1,000 | 36.44 | 20.15 |  |
| **This work** | **gDPP-T/pC6FA** | **Passivation** | **Vertical** | **73.7** | **1.5**×**10^6^** | **1,000** | **6.52** | **20.9** | **21.4** | | **5.4×10^4^** | **1,000** | **85.9** | **6.4** |  |

**Figure S1.** Overall fabrication process of FPA-vOECT.

**Figure S2.** Evaluation of pC6FA-passivation thickness to determine the optimized layer thickness for vOECT performance.

**Figure S3.** (a) Optimized iCVD process conditions for pC6FA synthesis and (b) Fourier-transform infrared (FT-IR) spectrum of the synthesized pC6FA film.

**Figure S4.** Quantitative EDS line-scan analysis of the domain–matrix structure in the FPA-vOECT channel. Cross-sectional TEM and EDS elemental mapping images show the spatial distribution of fluorine and sulfur in the gDPP-T/pC6FA channel region. The line-scan profile obtained from the yellow-boxed region reveals an increased fluorine atomic ratio accompanied by a decreased sulfur atomic ratio in the pC6FA-rich domain, confirming the formation of fluorine-rich domains embedded within the gDPP-T matrix.

**Figure S5.** Time-of-flight secondary ion mass spectrometry (ToF-SIMS) depth profile of the iCVD-treated vOECT, showing the spatial distribution of fluorine and sulfur.

**Figure S6.** Uniformity analysis of the iCVD vapor infiltration and thermal annealing process. (a) Photograph of eight FPA-vOECT devices fabricated on a single 3.5 cm × 4 cm glass substrate. The red boxes indicate the three randomly selected device positions used for cross-sectional TEM analysis. (Scale bar: 1 cm) (b) Cross-sectional TEM images of the channel regions obtained from positions #1, #2, and #3, showing the reproducible formation of the gDPP-T/pC6FA domain–matrix nanostructure across different devices on the same substrate (scale bar: 100 nm).

**Figure S7.** (a) Peak positions of the (100) lamellar stacking and (010) π-π stacking reflections, and (b) corresponding full width at half maximum (FWHM) values extracted from the out-of-plane and in-plane GIWAXS 1D line-cut profiles. (c) Comparison of the GIWAXS patterns between thermally annealed pristine gDPP-T and thermally annealed pC6FA-passivated gDPP-T films.

**Figure S8.** Atomic force microscopy (AFM) analysis of iCVD-passivated vOECT. (a) Pristine gDPP-T film after annealing, (b) pC6FA-passivated gDPP-T film after annealing.

**Figure S9.** Comparison of performance enhancement under different pC6FA passivation and thermal annealing conditions. Transconductance (*g*_m_) of the vOECT was measured under p-type operation for (a) pristine-vOECT without thermal annealing, (b) pristine-vOECT with thermal annealing, (c) FPA-vOECT without thermal annealing, and (d) FPA-vOECT with thermal annealing.

**Figure S10.** Comparison of performance enhancement achieved by different iCVD polymer passivation layers. (a) Transconductance (*g*_m_) comparison of the vOECT before and after iCVD passivation under p-type operation, and (b) molecular structures of the respective polymer materials.

**Figure S11.** Evolution of cyclic voltammetry (CV) curves during repeated potential cycling for pristine gDPP-T and pC6FA-passivated gDPP-T films in 0.1 M KCl aqueous electrolyte (vs Ag/AgCl), with measurements repeated three times.

**Figure S12.** Ultraviolet-visible (UV–Vis) absorbance changes relative to 0 V in 0.1 M KCl aqueous electrolyte for (a) pristine gDPP-T under oxidation, (b) pC6FA-passivated gDPP-T under oxidation, (c) pristine gDPP-T under reduction, and (d) pC6FA-passivated gDPP-T under reduction.

**Figure S13.** (a) Schematic illustration of the ambipolar OECT-based bipolar inverter. (b) Voltage transfer characteristics (VTCs) of the iCVD-passivated bipolar inverter measured under supply voltages |*V*_DD_| = 0.6 to 0.9 V. (c) Corresponding voltage gain |*V*_out_/*V*_in_| extracted from the VTCs.

**Figure S14.** Long-term operation of the inverter response over 40 cycles to input voltage square pulses is shown for (a) an n-mode inverter with *V*_DD_ ranging from 0.4 to 0.9 V and (c) a p-mode inverter with *V*_DD_ ranging from −0.1 to −0.6 V. (b,d) The transient responses of signal inversion were fitted with exponential functions to extract the characteristic time constants.

References

1. W. Huang, J. Chen, Y. Yao*, et al.* "Vertical Organic Electrochemical Transistors for Complementary Circuits." *Nature* 613, no. 7944 (2023): 496-502.

2. S. Wang, Y. Wang, X. Cai*, et al.* "A High-Frequency Artificial Nerve Based on Homogeneously Integrated Organic Electrochemical Transistors." *Nature Electronics* 8, no. 3 (2025): 254-66.

3. V. N. Le, K. N. Baustert, M. R. Brown*, et al.* "Improved Organic Electrochemical Transistor Stability Using Solvent Degassing and Chemical Doping." *Nature Electronics* 8, no. 2 (2025): 116-26.

4. E. A. de Moura, M. Luginieski, J. P. Serbena, and K. F. Seidel. "On–Off Ratio Improvement in Organic Electrochemical Transistors from Addition of a Pmma Layer at the Electrolyte Dielectric/Semiconductor Interface." *Journal of Applied Physics* 129, no. 15 (2021).

5. D. Ohayon, L. Q. Flagg, A. Giugni*, et al.* "Salts as Additives: A Route to Improve Performance and Stability of N-Type Organic Electrochemical Transistors." *ACS Materials Au* 3, no. 3 (2023): 242-54.

6. D. C. T. Nguyen, Q. Vu Thi, Q. H. Nguyen*, et al.* "Engineering Flexible Dopamine Biosensors: Blended Egylated Conjugated and Radical Polymers in Organic Electrochemical Transistors." *npj Flexible Electronics* 9, no. 1 (2025): 35.

7. E. Zeglio, Y. Wang, S. Jain*, et al.* "Mixing Insulating Commodity Polymers with Semiconducting N‐Type Polymers Enables High‐Performance Electrochemical Transistors." *Advanced Materials* 36, no. 23 (2024): 2302624.

8. Y. Lai, J. Cheng, M. Xie*, et al.* "Precisely Patterned Channels in a Vertical Organic Electrochemical Transistor with a Diazirine Photo‐Crosslinker." *Angewandte Chemie International Edition* 63, no. 18 (2024): e202401773.

9. X. Wu, T. L. D. Tam, S. Chen*, et al.* "All‐Polymer Bulk‐Heterojunction Organic Electrochemical Transistors with Balanced Ionic and Electronic Transport." *Advanced Materials* 34, no. 42 (2022): 2206118.

10. E. Stein, O. Nahor, M. Stolov*, et al.* "Ambipolar Blend-Based Organic Electrochemical Transistors and Inverters." *Nature communications* 13, no. 1 (2022): 5548.

11. N. Moscovich, S. Simotko, E. Reyn, I. Zerachia, A. Hadar, and G. L. Frey. "Balanced Ambipolar Oects through Tunability of Blend Microstructure." *ACS Applied Materials & Interfaces* 17, no. 30 (2025): 43327-38.

12. J. J. Samuel, A. Garudapalli, A. A. Mohapatra, C. Gangadharappa, S. Patil, and N. P. B. Aetukuri. "Single‐Component Cmos‐Like Logic Using Diketopyrrolopyrrole‐Based Ambipolar Organic Electrochemical Transistors." *Advanced Functional Materials* 31, no. 45 (2021): 2102903.

13. L. Lan, Y. Wang, X. Zhu, I. McCulloch, and W. Yue. "Ultrathin‐Film Small Molecule Mixed Conductors Exhibiting Ion‐Tunable Ambipolarity for High‐Performance Organic Electrochemical Transistors and Multivalued Logic Inverters." *Advanced Materials* 37, no. 35 (2025): 2501041.

14. Y. Zhang, E. R. van Doremaele, G. Ye*, et al.* "Adaptive Biosensing and Neuromorphic Classification Based on an Ambipolar Organic Mixed Ionic–Electronic Conductor." *Advanced Materials* 34, no. 20 (2022): 2200393.

15. T. Pan, X. Wu, M. Liu*, et al.* "Ambipolar Organic Bulk Heterojunction Electrochemical Transistors for High Gain Inverters." *ACS Materials Letters* 8, no. 1 (2025): 171-78.

16. G. Qi, M. Wang, S. Wang*, et al.* "High‐Performance, Single‐Component Ambipolar Organic Electrochemical Transistors with Balanced N/P‐Type Properties for Inverter and Biosensor Applications." *Advanced Functional Materials* 35, no. 2 (2025): 2413112.

17. S. Cong, J. Chen, M. Xie*, et al.* "Single Ambipolar Oect–Based Inverter with Volatility and Nonvolatility on Demand." *Science Advances* 10, no. 41 (2024): eadq9405.

18. R. B. Rashid, W. Du, S. Griggs, I. P. Maria, I. McCulloch, and J. Rivnay. "Ambipolar Inverters Based on Cofacial Vertical Organic Electrochemical Transistor Pairs for Biosignal Amplification." *Science advances* 7, no. 37 (2021): eabh1055.

19. Y. Wang, J. Tan, H. Hou*, et al.* "High Performing Ambipolar Organic Electrochemical Transistors and Solid‐State Inverters Enabled by Hydrophilic/Hydrophobic Side Chains Integration." *Advanced Materials*  (2025): e15186.

20. J. Li, Z. Li, J. Huang*, et al.* "High‐Performance Ambipolar Organic Electrochemical Transistors Based on Diketopyrrolopyrrole‐Dialkoxybithiazole Conjugated Polymers for Single‐Component Inverters." *Advanced Science*  (2026): e20003.

21. L. Shi, H. Yang, H. Li*, et al.* "Controlling Ambipolar Oect Threshold Voltage through Acceptor Unit Engineering of Conjugated Polymers." *Journal of Materials Chemistry C* 13, no. 37 (2025): 19437-43.

22. G.-Y. Ge, J. Xu, X. Wang*, et al.* "On-Site Biosignal Amplification Using a Single High-Spin Conjugated Polymer." *Nature Communications* 16, no. 1 (2025): 396.

23. Y. Sun, Y. Lan, M. Li*, et al.* "Indacenodithiophene‐Based Single‐Component Ambipolar Polymer for High‐Performance Vertical Organic Electrochemical Transistors and Inverters." *Aggregate* 5, no. 5 (2024): e577.
